# The Prevalence and Patterns of Toxicity With Immune Checkpoint Inhibitors in Solid Tumors: A Real-World Experience From a Tertiary Care Center in Oman

**DOI:** 10.7759/cureus.47050

**Published:** 2023-10-15

**Authors:** Bushra Salman, Nameer M AlWard, Zamzam Al-Hashami, Hadil Al-Sharqi, Hasan Al-Sayegh, Ikram A Burney

**Affiliations:** 1 Pharmacy Department, Sultan Qaboos Comprehensive Cancer Care and Research Centre, Muscat, OMN; 2 Oncology Department, Sultan Qaboos Comprehensive Cancer Care and Research Centre, Muscat, OMN; 3 Research Department, Sultan Qaboos Comprehensive Cancer Care and Research Centre, Muscat, OMN

**Keywords:** middle east, immune checkpoint inhibitors, oncology, cancer, sultanate of oman, immune-related adverse effects

## Abstract

Introduction

Immune checkpoint inhibitors (ICIs) have revolutionized the management of multiple cancers over the last decade. They work by employing the immune system and exhibiting activity over T cells resulting in immune upregulation. Despite their widespread use, they produce side effects that can limit their use. The immune-related adverse events (irAEs) can be sometimes significant. The irAEs caused by ICIs may occur at any time during the treatment and can vary in grade (G). We sought to study the prevalence and toxicity patterns of ICIs in Oman.

Methods

One hundred forty-one adult patients (≥18 years) who received at least one dose of nivolumab, pembrolizumab, atezolizumab, or durvalumab between 2016 and 2022 were included. The data were analyzed retrospectively using univariable and multiple-variable logistic regressions. The Wilcoxon rank-sum test and Cochran-Armitage trend test were also used to summarize the continuous and ordinal data.

Results

Out of the 141 patients, 80 patients (56.7%) received pembrolizumab, and 48 (34%) received nivolumab. Common irAEs included endocrine abnormalities, pneumonitis, and colitis. Thirty patients (21.3%) experienced varying irAE grade toxicity. Out of the 30, 23 patients (82%) developed grade 2 and 3 irAEs.

Discussion

Predictive analysis showed that male sex and lower hemoglobin (Hb) and bilirubin levels were all significant predictors (p < 0.05) when associated with irAE occurrence. The prevalence of irAEs was similar compared to other reports, literature reviews, or meta-analyses. Female sex has been mentioned previously also to be a predictive factor for endocrine-related toxicities.

## Introduction

Immune checkpoint inhibitors (ICIs) have revolutionized the treatment of various malignancies over the last few years. Monoclonal antibodies targeting cytotoxic T lymphocyte-associated antigen 4 (CTLA4) and the programmed death-1 (PD-1) receptor and its ligand programmed death-ligand 1 (PD-L1) have been approved as the standard of care in different tumor sites. Under normal circumstances, the immune checks serve as antigen non-specific inhibitory signals to prevent the overactivation of the immune system. However, during cancer development, they are thought to be the main pathway through which malignant cells escape immune surveillance and apoptosis [1]. Thereby, the efficacy of these targeted agents lies in their ability to “release the brakes” on the blocked T cells resulting in immune upregulation. Studies have shown their efficacy in a wide range of solid tumors and in different treatment settings with sustained long-term remission and prolonged survival [2].

Inherent within their pharmacological action is the drawback of causing significant toxicities and autoimmune side effects, the so-called immune-related adverse events (irAEs) [3]. These irAEs may involve several organs from different body systems, and although most irAEs are generally mild in intensity, serious, irreversible, or even fatal adverse reactions sometimes occur [4]. The frequency of irAEs for different ICIs was found to be in the range of 60%-85%, mostly grades (G) 1 and 2. Grade 3 or higher irAEs were found to occur in approximately 10%-20% of the patients [5]. In general, high-grade toxicities are less common from anti-PD-1 (nivolumab or pembrolizumab) than for the CTLA4 inhibitors (ipilimumab) [1].

The onset of these adverse reactions can vary from the first few weeks to months after starting the treatment [4]. They can potentially occur at any time, even after discontinuing the treatment. Dermatological irAEs are usually the first to manifest at around 2-4 weeks following treatment initiation, followed by gastrointestinal (GI) and hepatic AEs that occur around the sixth and seventh weeks following treatment. Endocrine abnormalities usually occur at 2-3 months and may continue following treatment cessation. Pneumonitis also has a similar time frame of 2-3 months but can occur as early as nine days and up to 24 months. However, late toxicity with immune checkpoint inhibitors (ICIs) is less characterized than early toxicity [6].

It is unknown which patients are more likely to experience severe irAEs, and the current incidences and characteristics are mainly reported from large perspective clinical trials where patients are closely monitored. However, patients treated outside clinical trials may have different clinical characteristics and monitoring patterns, leading to different outcomes. Furthermore, the selection of patients who are likely to have optimal response to immunotherapy has been the interest of many clinical studies. Programmed death-ligand 1 (PD-L1) expression, microsatellite instability (MSI), and tumor mutational burden (TMB) are biomarkers that have been studied and approved to predict response to ICI. There is a growing body of evidence suggesting that irAE may predict response to ICI across various tumor sites. Studies have reported marked improvements in response rate, progression-free survival, and overall survival in patients who experienced irAE related to anti-PD-1 or anti-PD-L1 antibodies [7]. However, it is not clear whether the type of ICIs, irAE time of onset, severity, and site influence ICI efficacy. There are currently only a few studies describing the toxicity profile of these agents and its influence on ICI efficacy. Therefore, the aim of this study is to evaluate the real-world data of the prevalence and patterns of irAEs in patients with solid-organ malignancies treated with PD-1 and its ligand PD-L1 blockade.

## Materials and methods

This was a retrospective analysis of all patients with solid cancers who received at least one dose of nivolumab, pembrolizumab, atezolizumab, or durvalumab at the Sultan Qaboos University Hospital (SQUH) and Sultan Qaboos Comprehensive Cancer Care and Research Centre (SQCCCRC) from January 2016 to August 2022. These agents were administered according to the National Comprehensive Cancer Network (NCCN®), British Columbia Cancer Agency (BCCA®), and National Health Service (NHS®) guidelines. The drugs were administered and continued until disease progression or intolerable toxicities were evident. Toxicities were assessed by the Common Terminology Criteria for Adverse Events (CTCAE) version 5.0 and the American Society of Clinical Oncology (ASCO) clinical practice guidelines on the management of immune-related adverse events (AEs) in patients treated with ICIs (2021) [8]. Clinical follow-up was performed after the completion of each cycle. Radiological evaluation was performed by computed tomography or positron emission tomography/computed tomography scans as per the institutional policies after 3-4 cycles and when clinically indicated subsequently.

Inclusion and exclusion criteria

Consecutive adult patients (>18 years) who received at least one cycle of ICI, as part of their treatment protocol, were included. ICIs were administered as a single agent or in combination with chemotherapy or other targeted therapies, according to the abovementioned guidelines.

Data collection

The study data were extracted retrospectively from the SQCCCRC and SQUH electronic records and health information systems. Data were collected from the patient’s first visit until the last follow-up date. Data including patients’ demographics, body mass index (BMI), cancer type, hematological and biochemical laboratory test results at the time of ICI initiation, disease history including cancer stage, prior therapies, details of current therapy, and drug-related AEs and irAEs (e.g., occurrence, CTCAE grade, laboratory tests, type, and management) were also collected. Comorbidities were collected retrospectively from patient records to calculate the Charlson Comorbidity Index (CCI), which is a well-validated and easily applicable approach for predicting long-term prognosis and survival due to comorbid conditions. This study was conducted with SQCCCRC Institutional Review Board and Ethics Committee approval (CCCRC-29-2022).

Statistics

Continuous variables were described using medians and ranges and the categorical variables using frequencies and percentages. The nivolumab and pembrolizumab groups were compared using the Wilcoxon rank-sum test for the continuous data, the Cochran-Armitage trend test for the ordinal data, and the Fisher exact test for other categorical data. Univariable logistic regression models were used to examine predictive factors’ association with ICI toxicity; the Firth method was used for the analyses with low frequencies. Odds ratios (OR) with 95% confidence intervals (CI) were reported from each logistic regression model. A multivariable regression model was constructed using all significant predictors from the univariable analyses and the baseline laboratory measurements. The number of patients with available data was reported for each analysis. P-values of ≤0.05 were considered statistically significant. The Statistical Analysis System (SAS) software version 9.4 (SAS Institute Inc., Cary, NC) was used for all statistical analyses.

## Results

One hundred forty-one patients received ICIs between 2016 and 2022. The median age at diagnosis was 54 years (range = 22-85); 80 patients (56.7% of the study population) were male (Tables 1, 2). The most common primary tumor site was the lung (n = 34, 24.1%), followed by the breast (n = 21, 14.9%), head and neck (n = 16, 11.3%), kidney (n = 13, 9.2%), and stomach (n = 12, 8.5%). Most patients had stage IV disease at the time of ICI initiation (n = 118, 85.5%). Thirty-three patients (23.4%) had a smoking history.

**Table 1:** Baseline characteristics of the overall study population and nivolumab and pembrolizumab cohorts.

	Overall (N = 141) (n {%})	Nivolumab (N = 48) (n {%})	Pembrolizumab (N = 80) (n {%})
Age	N = 141	N = 48	N = 80
<65 years	111 (78.7)	43 (89.6)	62 (77.5)
≥65 years	30 (21.3)	5 (10.4)	18 (22.5)
Gender	N = 141	N = 48	N = 80
Female	61 (43.3)	18 (37.5)	40 (50)
Male	80 (56.7)	30 (62.5)	40 (50)
Primary site	N = 141	N = 48	N = 80
Lung	34 (24.1)	10 (20.8)	19 (23.8)
Breast	21 (14.9)	0 (0)	21 (26.3)
Head and neck	16 (11.3)	6 (4.7)	10 (7.8)
Kidney	13 (9.2)	8 (16.7)	5 (6.3)
Stomach	12 (8.5)	6 (12.5)	6 (7.5)
Others	61 (43.3)	24 (50)	29 (36.3)
Smoking history	N = 141	N = 48	N = 80
No	108 (76.6)	41 (85.4)	60 (75)
Yes	33 (23.4)	7 (14.6)	20 (25)
Immunotherapy starting year	N = 141	N = 48	N = 80
2016	5 (3.6)	4 (8.3)	1 (1.3)
2017	6 (4.3)	5 (10.4)	1 (1.3)
2018	6 (4.3)	0 (0)	6 (7.5)
2019	26 (18.4)	13 (27.1)	12 (15)
2020	21 (14.9)	8 (16.7)	11 (13.8)
2021	26 (18.4)	4 (8.3)	16 (20)
2022	51 (36.2)	14 (29.2)	33 (41.3)
Stage	N = 138	N = 46	N = 80
II	6 (4.3)	1 (2.2)	5 (6.3)
III	14 (10.1)	2 (4.3)	10 (12.5)
IV	118 (85.5)	43 (93.5)	65 (81.3)
Number of metastasis sites	N = 141	N = 48	N = 80
None	22 (15.6)	4 (8.3)	15 (18.8)
1	43 (30.5)	11 (22.9)	28 (35)
2	39 (27.7)	16 (33.3)	20 (25)
3+	37 (26.2)	17 (35.4)	17 (21.3)
Previous chemotherapy	N = 140	N = 48	N = 79
No	54 (38.6)	15 (31.3)	29 (36.7)
Yes	86 (61.4)	33 (68.8)	50 (63.3)
Previous radiotherapy	N = 141	N = 48	N = 80
No	86 (61)	30 (62.5)	51 (63.8)
Yes	55 (39)	18 (37.5)	29 (36.3)
Concomitant with chemotherapy	N = 141	N = 48	N = 80
No	93 (66)	41 (85.4)	43 (53.8)
Yes	48 (34)	7 (14.6)	37 (46.3)

**Table 2:** Age, BMI, comorbidity score**, and laboratory measurements of the study population and pembrolizumab and nivolumab cohorts. *Comparing the nivolumab and pembrolizumab groups using Wilcoxon rank-sum tests. **Comorbidity score using the Charlson Comorbidity Index (CCI). BMI, body mass index; Hb, hemoglobin; WBC, white blood cells; ANC, absolute neutrophil count; ALT, alanine transaminase; AST, aspartate transaminase, TSH, thyroid-stimulating hormone; T4, thyroxine

	N	Study population	N	Nivolumab	N	Pembrolizumab	P-value*
		Median (range)		Median (range)		Median (range)	
Age (year)	141	54 (22-85)	48	54.5 (28-76)	80	52.5 (22-85)	0.64
BMI	141	22.9 (13.2-54.3)	48	22.2 (15.6-34.5)	80	23.3 (13.2-45.1)	0.27
Comorbidity score	141	7 (2-12)	48	7 (2-12)	80	7 (2-12)	0.54
Hb (g/dL)	141	10.8 (7.1-19.7)	48	10.5 (7.1-19.7)	80	10.7 (7.3-16.2)	0.86
Platelet (10^9^/L)	141	293 (10.9-776)	48	320.5 (10.9-776)	80	284 (26.1-721)	0.87
WBC (10^9^/L)	141	6.1 (2.3-40.4)	48	6.3 (2.3-14.3)	80	6 (2.4-40.4)	0.91
ANC (10^9^/L)	141	3.7 (0.7-39.9)	48	3.7 (0.9-11.7)	80	3.3 (0.7-39.9)	0.97
ALT (IU/L)	140	15.2 (4-120.7)	47	15 (4-111.2)	80	15 (5.1-111)	0.72
AST (IU/L)	137	18 (8-286.8)	47	17 (8-176)	77	19.9 (9.3-286.8)	0.34
Bilirubin (µmol/L)	140	5.3 (2.3-160.3)	48	5 (2.3-160.3)	79	5.3 (3-79.9)	0.67
Creatinine (µmol/L)	140	59 (3.8-374)	47	59 (26-374)	80	58 (25-176)	0.73
Cortisol (nmol/L)	101	336.5 (2.6-3394)	34	378.4 (16-660)	56	320.8 (2.6-3394)	0.47
TSH (mIU/L)	132	2.1 (0-141.8)	46	1.8 (0.3-45.5)	75	2.4 (0-141.8)	0.2
T4 (pmol/L)	121	15.7 (1.7-34.2)	44	15.1 (8.9-34.2)	69	15.8 (1.7-32.3)	0.15

Eighty patients (56.7%) received pembrolizumab, 48 (34%) patients received nivolumab, and nine (6.4%) and four (2.8%) patients received atezolizumab and durvalumab, respectively. Forty-eight (34%) patients received concomitant chemotherapy, and 86 (61.4%) had received prior chemotherapy. Patients receiving pembrolizumab were more likely to receive concomitant chemotherapy compared to those who received nivolumab (37 {46.3%} versus seven {14.6%}, p-value = 0.0002). All patients with breast cancer (n = 21) received pembrolizumab. Laboratory results are presented in Table 2. Apart from concomitant chemotherapy, there were no statistically significant differences in the laboratory test values between patients who received nivolumab or pembrolizumab.

Thirty patients (21.3%) experienced 40 toxicities (adverse events) after starting ICI treatment. Of these 30 patients, 28 had data available with regard to toxicity grade; 14 (50%) had maximum grade 2 toxicity, and nine (32%) had maximum grade 3 toxicity. Two (7%) patients had maximum grade 4 toxicity, and two (7%) other patients had grade 5 toxicity. Outcome data are available for 25 of these 30 patients; most patients discontinued treatment (16/25; 64%). Seven patients (28%) required close monitoring, and two (8%) had to have the treatment delayed (Table 3). All patients who experienced any toxicity grade of pneumonitis, hepatitis, and/or hematological toxicities discontinued the treatment. Similarly, for all but one patient experiencing colitis-related symptoms, ICI treatment was discontinued. Treatment was continued with close monitoring in six patients with grade 2 hypothyroidism and in one patient with lower limb weakness. Treatment was delayed for one patient with adrenal insufficiency (grade 1) and one patient with grade 2 hypothyroidism. Hospitalization was required in 19 patients (63%). All patients with pneumonitis received steroids, and improvement was seen in 6/8 patients. Two patients deteriorated and succumbed to death after developing immune-related pneumonitis. There were no statistically significant differences between the nivolumab and pembrolizumab groups with regard to toxicity prevalence, grade, and patients’ outcomes. The development of toxicity did not have a significant impact on the duration of treatment with ICI (median treatment duration in patients without toxicity was 3.2 months versus 4.4 months in patients who developed toxicity, p-value = 0.466).

**Table 3:** Number and grades of toxicity after immune checkpoint inhibitor treatment and patients’ outcomes. *Comparing the nivolumab and pembrolizumab groups using the Cochran-Armitage trend test for ordinal data and the Fisher exact test for other categorical data. **Toxicity grade data are available for 28 patients (toxicity grade information is unknown for two patients). ***P-value obtained using the Cochran-Armitage trend test. ****Outcome data are available for 25 patients (outcome information is unknown for five patients).

	Study population (n {%})	Nivolumab (n {%})	Pembrolizumab (n {%})	P-value*
Any toxicity	30/141 (21.3)	14/48 (29.2)	16/80 (20)	0.28
Pneumonitis	7/141 (5)	2/48 (4.2)	5/80 (6.3)	0.71
Hypothyroidism	9/141 (6.4)	5/48 (10.4)	4/80 (5)	0.29
Hepatitis/hepatic toxicity	3/141 (2.1)	1/48 (2.1)	2/80 (2.5)	1
Diarrhea/colitis	5/141 (3.5)	1/48 (2.1)	3/80 (3.8)	1
Adrenal insufficiency	3/141 (2.1)	2/48 (4.2)	1/80 (1.3)	0.56
Anemia/thrombocytopenia	5/141 (3.5)	1/48 (2.1)	2/80 (2.5)	1
Others	5/141 (3.5)	2/48 (4.2)	2/80 (2.5)	0.63
Toxicity maximum grade**				
Grade 1	1/28 (3.6)	1/12 (8.3)	0/16 (0)	0.13***
Grade 2	14/28 (50)	7/12 (58.3)	7/16 (43.8)	
Grade 3	9/28 (32.1)	3/12 (25)	6/16 (37.5)	
Grade 4	2/28 (7.1)	1/12 (8.3)	1/16 (6.3)	
Grade 5	2/28 (7.1)	0/12 (0)	2/16 (12.5)	
Toxicity outcome****				
Close monitoring	7/25 (28)	4/10 (40)	3/15 (20)	0.56
Delayed treatment	2/25 (8)	1/10 (10)	1/15 (6.7)	
Treatment discontinued	16/25 (64)	5/10 (50)	11/15 (73.3)	

Predictive logistic regression models suggest (Table 4) that females were less likely to develop toxicity after starting ICI therapy compared to males (OR = 0.32, 95% CI = 0.13-0.81, p = 0.016). Patients who received previous chemotherapy were at a higher risk of developing toxicities compared with patients with no previous chemotherapy history (OR = 3.1, 95% CI = 1.17-8.17, p = 0.023). Concomitant chemotherapy treatment was not a significant predictor of toxicity (p = 0.6). A higher hemoglobin (Hb) (g/dL) level was associated with lower chance of toxicity (OR = 0.78, 95% CI = 0.61-0.99, p = 0.04). Other factors were not significantly associated with ICI toxicity. Predictive logistic regression models were also executed separately for patients treated with nivolumab and pembrolizumab. In the nivolumab group, none of the tested predictive factors were significantly associated with toxicity. In the pembrolizumab group, male sex, lower Hb, higher alanine transaminase (ALT), and previous chemotherapy were associated with a higher risk of developing toxicity.

**Table 4:** Toxicity odds ratios (OR) and 95% confidence intervals (CI) for predictive factors using univariable logistic regression models. *Number of patients included in the model (patients with available data). **Using logistic regression analysis for binary outcomes. ***Using the Firth logistic regression method. BMI, body mass index; Hb, hemoglobin; WBC, white blood cells; ANC, absolute neutrophil count; ALT, alanine transaminase; AST, aspartate transaminase; TSH, thyroid-stimulating hormone; T4, thyroxine; ICI, immune checkpoint inhibitor

	All patients			Nivolumab			Pembrolizumab		
Predictive factor	N*	OR** (95% CI)	P-value **	N*	OR** (95% CI)	P-value	N*	OR** (95% CI)	P-value
Age (one year)	141	0.99 (0.96-1.02)	0.54	48	1 (0.94-1.05)	0.92	80	1 (0.96-1.03)	0.87
BMI	141	0.99 (0.9-1.05)	0.69	48	1.15 (1-1.32)	0.057	79	0.9 (0.85-1.04)	0.2
Sex (female versus male)	141	0.32 (0.13-0.81)	0.016	48	0.35 (0.08-1.47)	0.15	80	0.26 (0.08-0.89)	0.032
Diagnosis year (one year)	138	0.9 (0.8-1.02)	0.089	47	1.01 (0.79-1.29)	0.92	78	0.9 (0.78-1.04)	0.14
Smoking history (yes versus no)	141	1.25 (0.5-3.15)	0.63	48	2.05 (0.39-10.64)	0.39	80	1.49 (0.45-4.96)	0.52
Stage (4 versus 2-3)	138	6.19 (0.79-48.28)	0.082	46	0.87 (0.07-10.42)	0.91	80	10.33 (0.53-199.82)	0.12
Number of metastasis sites	141			48			80		
1 versus none		6.36 (0.76-53.39)	0.088		1.71 (0.13-22.51)	0.68		8.96 (0.43-187.4)	0.16
2 versus none		8.25 (0.99-68.98)	0.052		1 (0.08-12.56)	1		17.22 (0.82-363.22)	0.067
3+ versus none		5.79 (0.67-49.89)	0.11		1.25 (0.1-15.11)	0.86		7.48 (0.32-174.03)	0.21
Comorbidity score	141	1.13 (0.93-1.37)	0.23	48	1.09 (0.75-1.59)	0.19	80	1.22 (0.94-1.58)	0.14
Chemotherapy	140			48			79		
Concomitant use with ICI versus not used previously		0.76 (0.13-4.59)	0.77		2.25 (0.11-45.72)	0.6		3 (0.1-89.63)***	0.53
Previously used versus not used concomitantly with ICI		2.87 (0.88-9.39)	0.08		0.9 (0.21-3.78)	0.89		13.45 (0.66-276.15)***	0.09
Concomitant use with ICI and previously used versus none		2.67 (0.69-10.39)	0.16		0.56 (0.05-6.77)	0.65		12.17 (0.57-261.1)***	0.11
Previously used chemotherapy (yes versus no)	140	3.1 (1.17-8.17)	0.023	48	0.75 (0.2-2.8)	0.67	79	11.99 (1.49-96.37)	0.02
Previous radiotherapy (yes versus no)	141	0.73 (0.31-1.71)	0.47	48	0.57 (0.15-2.2)	0.42	80	1.07 (0.34-3.32)	0.91
Concomitant chemotherapy (yes versus no)	141	0.79 (0.33-1.89)	0.6	48	0.97 (0.16-5.69)	0.97	80	0.88 (0.29-2.66)	0.82
Hb (g/dL)	141	0.78 (0.61-0.99)	0.043	48	0.93 (0.69-1.26)	0.65	80	0.68 (0.47-0.99)	0.042
Platelet (10^9^/L)	141	1.002 (0.999-1.005)	0.19	48	1.003 (0.999-1.01)	0.15	80	1.001 (0.997-1.004)	0.79
WBC (10^9^/L)	141	1 (0.93-1.08)	0.97	48	1.1 (0.92-1.32)	0.3	80	0.99 (0.9-1.08)	0.78
ANC (10^9^/L)	141	1 (0.92-1.08)	0.95	48	1.11 (0.91-1.35)	0.32	80	0.98 (0.88-1.09)	0.73
ALT (IU/L)	140	1 (0.98-1.02)	0.73	47	0.94 (0.88-1.02)	0.12	80	1.04 (1.001-1.07)	0.042
AST (IU/L)	137	0.99 (0.98-1.01)	0.51	47	0.98 (0.95-1.02)	0.31	77	1 (0.99-1.02)	0.78
Bilirubin (µmol/L)	140	0.94 (0.85-1.03)	0.19	48	0.94 (0.82-1.08)	0.38	79	0.94 (0.82-1.08)	0.37
Creatinine (µmol/L)	140	1 (0.99-1.01)	0.98	47	1 (0.99-1.01)	0.88	80	1 (0.98-1.02)	0.76
Cortisol (nmol/L)	101	1.001 (1-1.003)	0.08	34	0.999 (0.994-1.003)	0.58	56	1.001 (1-1.003)	0.11
TSH (mIU/L)	132	1.04 (0.98-1.09)	0.2	46	1.07 (0.95-1.19)	0.27	75	1.03 (0.98-1.09)	0.26
T4 (pmol/L)	121	1.01 (0.92-1.11)	0.88	44	1.08 (0.92-1.28)	0.34	69	0.95 (0.83-1.08)	0.42

In the multivariable logistic regression model, sex and hemoglobin levels remained statistically significant (Table 5), and bilirubin appeared to be statistically significant in the multivariable logistic regression opposite to the univariable analysis. Previous chemotherapy was not a significant predictor in the multivariable model when compared to the univariable model. This could be due to the smaller sample size (n = 90) and the low number of events (18 toxicities, 1.4 events per variable) included in this model.

**Table 5:** Toxicity odds ratios (OR) and 95% confidence intervals (CI) for selected predictive factors using multivariable logistic regression model (N = 90*). *Ninety patients have available data (complete case) and were included in the model. Hb, hemoglobin; WBC, white blood cells; ANC, absolute neutrophil count; ALT, alanine transaminase; AST, aspartate transaminase; TSH, thyroid-stimulating hormone; T4, thyroxine

Predictive factor	OR (95% CI)	P-value
Sex (female versus male)	0.15 (0.03-0.83)	0.03
Previous chemotherapy (yes versus no)	3.49 (0.53-22.81)	0.19
Hb (g/dL)	0.59 (0.35-0.99)	0.048
Platelet (10^9^/L)	0.999 (0.994-1.004)	0.7
WBC (10^9^/L)	1.54 (0.9-2.65)	0.12
ANC (10^9^/L)	0.58 (0.29-1.15)	0.12
ALT (IU/L)	1.01 (0.92-1.1)	0.89
AST (IU/L)	1.04 (0.99-1.1)	0.15
Bilirubin (µmol/L)	0.61 (0.39-0.95)	0.027
Creatinine (µmol/L)	0.98 (0.94-1.01)	0.22
Cortisol (nmol/L)	1.001 (0.999-1.004)	0.38
TSH (mIU/L)	1.03 (0.94-1.14)	0.48
T4 (pmol/L)	0.98 (0.81-1.17)	0.8

## Discussion

In this study, the prevalence and pattern of irAEs with ICI in Omani patients treated in a real-world setting were investigated. The prevalence of grade 1-5 irAEs was found to be around 21% with a mortality rate of 1.4%. The most commonly encountered toxicities were hypothyroidism, pneumonitis, and colitis. Male sex, low hemoglobin, and prior use of chemotherapy were found to predict the development of toxicity with ICIs, especially with pembrolizumab.

The introduction of ICIs into clinical practice has transformed the prognosis of patients with several types of cancers. However, significant morbidity due to irAEs results in compromised optimal outcomes. Studies have found a high rate of irAEs, with low-grade (grades 1-2) toxicities observed in more than 90% of patients, and more severe effects (grades 3-5) reported in 20%-60% of the subjects [9]. For example, more than 70% of lung cancer patients in the KEYNOTE-024 trial experienced any-grade toxicity, of which around 30% were grade 3 or higher [10]. Moreover, in the CheckMate-238 trial, adverse events were reported in 97% of the patients who received nivolumab [11]. Additionally, 65% of the patients with melanoma receiving single-agent anti-PD-1 inhibitors, both in adjuvant and metastatic settings, experienced toxicity [12].

On the contrary, we observed a relatively low rate of irAEs with ICI-based therapy, ranging from 20% for grade 2-3 toxicities to 2% for grade 4 irAEs. There were two ICI-related deaths in the cohort representing a rate of less than 2%. Toxicity rate did not differ significantly between patients receiving pembrolizumab (20%) and those on nivolumab (29%), although higher grades of toxicity were observed in the pembrolizumab group, which was also statistically not significant. In line with our findings, other authors also reported a lower rate of irAEs related to ICIs. For example, a meta-analysis by Wu et al. of 92 studies in patients with urological cancers reported an overall incidence of 34% for irAEs of all grades and 10% for irAEs of grade 3 and above [13].

Previous studies have hypothesized that the reason for this low incidence may be because of significant heterogeneity and unobserved confounders such as the disease site, concomitant therapies, and other concomitant medications [14]. Additionally, others considered irAEs to be influenced by many factors including, but not limited to, age, BMI, smoking, comorbidities, and performance status (PS) [15]. For example, age < 60 years and high BMI [16] can contribute to a higher rate of adverse events [15]. However, these reports have not been consistent [17]. In our cohort, patients were relatively young, with a median age of 54 years, and the median BMI was 23 kg/m^2^, and whether the ideal BMI contributed to a lower rate of irAEs despite the young age needs to be further investigated. Smoking history, significant comorbidities, and performance status have also been found to impact tolerance and survival with ICIs [15]. However, we had significant underreporting of smoking status and Eastern Cooperative Oncology Group (ECOG) performance status, and their impact on irAE occurrence cannot be fully elucidated. The use of concomitant chemotherapy was found to increase irAE rate and may also depend on the specific type of agent used. In our cohort, around 35% of the patients received concomitant chemo-immunotherapy. Furthermore, the use of steroid medications concomitantly with immunotherapy has been found to reduce its efficacy; however, there are no data to confirm that the concurrent use of steroid might also halt the development of irAEs. This is an area that needs more investigation. Nevertheless, in our cohort, the concurrent use of corticosteroids as a part of antiemetic protocol might have altered the prevalence of irAEs [18]. Most importantly, it is possible that the low rate of irAEs observed in our study is due to unrecognized grade 1 toxicity and/or the lack of documentation.

The most common irAEs seen were endocrine-related (6%), followed by pneumonitis (5%) and colitis (4%). However, skin rash was not a significant irAE in this cohort. In general, the prevalence of toxicity remained much lower than other studies [11]. For example, the treatment-related toxicities in patients with melanoma included thyroiditis in 29.4% of the patients, colitis in 27.6%, rash in 24.7%, hepatitis in 18.2%, arthritis in 13.5%, hypophysitis in 12.9%, pneumonitis in 8.8%, vitiligo-like depigmentation in 8.8%, and pancreatitis in 7.6%. However, in this study, around 30% of the patients received combination immunotherapy with anti-CTLA4/anti-PD-1 inhibitors, which have been linked to higher irAEs [12]. Also, endocrinopathies (mostly thyroiditis), pneumonitis, hepatitis, and colitis were reported in up to 20% of the patients in large clinical trials [1]. Pneumonitis was the second most prevalent irAE in our series, which reflects our patient population with lung cancer representing 24%. Multiple studies have reported that higher proportion of lung cancer patients develops pneumonitis, whereas cutaneous toxicity is higher in patients with melanoma [19]. ICI-related pneumonitis has an incidence of 3%-5% [20], leading to mortality in around 10% of the affected cases [21].

This lack of specific predictors of irAEs raises a significant clinical challenge, as the search for predictive biomarkers continues. Multiple specific and non-specific predictive biomarkers have been suggested; however, they are not yet considered relevant in clinical practice guidelines [15]. As shown in the univariable regression analysis (Table 4), some clinical findings such as sex, previous chemotherapy use, and hemoglobin level predicted toxicities in our study. Female sex was associated with a lower risk of experiencing irAEs (4/40 females and 12/39 males developed irAEs), which remained significant even in the univariable regression model. This is in variance to a recent study showing that the six-month cumulative incidence of grade (G) ≥ 2 irAEs was significantly higher in females (61.4% {95% CI = 41.0-91.9}) compared to males (27.9% {95% CI = 16.8-46.2%}) (p = 0.005) [22]. Other studies have shown that sex can impact the type of irAEs with females being more prone to experience thyroid- and endocrine-related toxicities compared to males [23] and males being more prone to experiencing hypophysitis [24] and pulmonary toxicity with PD-1/PD-L1 inhibitors [15].

Another important factor, which is prior chemotherapy, was found to increase the probability of experiencing irAEs as shown in other studies [15]. However, this difference was not significant in the multivariable regression, probably due to small sample size. It is speculated that chemotherapy affects the tumor microenvironment, as well as the adaptive immune response, by increasing the abundance of intra-tumoral cluster of differentiation 8+ (CD8+) T cells, which enhances the effect (and probably the side effects) of ICIs [25]. Surprisingly, we found that Hb level at the time of the initiation of ICI was also shown to be a significant risk factor in this study, a finding not reported previously. The effect of other blood cells in this regard is more studied, such as the neutrophil-to-lymphocyte ratio (NLR) [26]. Nevertheless, Becker et al. found that an Hb level of <11 g/dL is strongly associated with tumor hypoxia, a factor that was found to negatively impact therapeutic outcomes in patients with solid tumors [27]. How this affects response and toxicity to ICI needs further investigation. Other molecular biomarkers have also been explored to find a correlation with immune-related toxicities. One such marker known as the microRNA pathway-based biomarker marker was suggested to be sensitive in predicting irAEs, providing 80% accuracy in predicting grade 2 and higher toxicities [28]. All these efforts are undertaken with the goal of optimizing and personalizing treatment with ICIs to achieve maximum benefit.

In our cohort, 64% of patients experiencing irAEs (73% with pembrolizumab and 50% with nivolumab) had their treatment discontinued, representing 11% of the total cohort. This rate of discontinuation is similar to other studies, where a 13% discontinuation rate in patients with advanced melanoma was reported [29]. Only 8% experienced treatment delays, and in around 28%, treatment was continued with close monitoring. All patients with pneumonitis, colitis, hepatitis, and hematological toxicities had treatment discontinuation. In our cohort, out of the two patients who were reintroduced on ICI after a delay period, both were able to continue further doses with grade 1 or lower toxicity. The development of toxicity was not associated with a shorter duration of treatment as one would expect; on the contrary, the median duration was numerically higher, but this was not statistically significant. However, this might reflect the better treatment response in patients who experience any degree of irAEs, which was documented in many studies [30].

Our study has some limitations. We believe that toxicities lower than grade 3 were under reported and do not reflect the true magnitude of irAEs, as is often the case with retrospective studies. Additionally, data about the line of therapy upon initiating ICI in advanced stages were lacking, and the low median duration of treatment with ICI in our study might be related to the late initiation of ICIs. However, this needs to be further investigated. Moreover, the sample size was small. However, the experience with ICI in Oman is still evolving, and the results of this study may serve as a benchmark.

## Conclusions

This study has shown the ICIs are generally well-tolerated in the Omani population, with a low rate of toxicity. The most common toxicities noted were similar to those reported in other studies, such as endocrine abnormalities, pneumonitis, and colitis. Immune-related death was also similar to other reported studies at a rate of 1%-2%.

## Appendices

Table 6 shows the age distribution in the study population and the comparison groups.

**Table 6:** Age distribution in the study population and the comparison groups.

	Study population (N = 141)	Nivolumab (N = 48)	Pembrolizumab (N = 80)
20-<30 years	10 (7.09)	1 (2.08)	9 (11.25)
30-<40 years	24 (17.02)	9 (18.75)	15 (18.75)
40-<50 years	19 (13.48)	6 (12.5)	12 (15)
50-<60 years	38 (26.95)	19 (39.58)	17 (21.25)
60-<70 years	36 (25.53)	11 (22.92)	17 (21.25)
70+ years	14 (9.93)	2 (4.17)	10 (12.5)
